# Spatial and host-specific structuring in symbiont community composition of an endemic Hawaiian octocoral, *Sarcothelia edmondsoni* (Verrill 1928)

**DOI:** 10.7717/peerj.20549

**Published:** 2026-01-13

**Authors:** Erika M. Cabell, Cynthia L. Hunter

**Affiliations:** Marine Biology Graduate Program, School of Life Sciences, University of Hawaii at Manoa, Honolulu, HI, United States of America

**Keywords:** Symbiodinaceae, SymPortal, Octocoral, Symbiosis, Ecotype, Morphotypes, ITS2, Endemic

## Abstract

Coral reefs are increasingly threatened by climate-induced bleaching, yet some taxa—like the Hawaiian endemic octocoral *Sarcothelia edmondsoni*—exhibit notable stress tolerance. This study investigates whether distinct color morphotypes (blue and brown) of *S. edmondsoni* maintain stable or flexible symbiont associations that might underlie this resilience. Using high-throughput ITS2 sequencing and SymPortal analyses, we characterized Symbiodiniaceae communities across morphotypes on three Hawaiian Islands. Assemblages were overwhelmingly dominated (>99%) by *Symbiodinium* (Clade A), particularly *S. tridacnidorum* (ITS2 type A3), with blue morphotypes consistently hosting more diverse symbiont profiles. Dinoflagellate community composition varied significantly by morphotype and location, with no ITS2 profile shared across all morphotype–island combinations. Bray–Curtis analyses revealed strong ecological structuring, while UniFrac (a measure of evolutionary relatedness) indicated phylogenetic similarity, suggesting intragenomic or ecotypic divergence within a conserved lineage. Morphotype-specific associations may reflect environmental adaptation or host–symbiont specificity. The greater symbiont diversity in blue morphotypes, coupled with the lack of profile overlap among sites, points to fine-scale host–symbiont structuring shaped by local environmental conditions. These results demonstrate that *Sarcothelia edmondsoni* hosts morphotype- and location-specific Symbiodiniaceae communities within a conserved lineage, revealing fine-scale ecological structuring and potential symbiont ecotypes that may contribute to this species’ resilience across variable reef environments. This study supports previous findings that symbiont community structure is shaped by the combined influence of host specificity and local environmental conditions.

## Introduction

Dinoflagellates in the family *Symbiodiniaceae* form a highly diverse lineage of unicellular algae that includes both free-living taxa and symbiotic forms, commonly referred to as “zooxanthellae” (LaJeunesse et al., 2018). These symbionts form mutualistic associations with a wide range of marine invertebrates—including corals, anemones, jellyfish, clams, foraminifera, flatworms, sponges, and ciliates (Trench, 1993). Zooxanthellate corals are energetically dependent on their symbionts, which can translocate up to 95% of photosynthetically fixed carbon to the host, covering as much as 90% of the coral’s daily energy needs (Wooldridge, 2010; Fournier, 2013; Titlyanov & Titlyanova, 2020). In exchange, corals provide the algae with inorganic nutrients and structural refuge from predators and environmental disturbances (Wooldridge, 2010). The advent of PCR-based techniques has revealed varying levels of host-symbiont specificity across ecological and geographic gradients (Rowan & Knowlton, 1995; LaJeunesse et al., 2010a; Cooper et al., 2011; De Souza et al., 2022; Ng et al., 2024).

The identity of a coral’s symbionts is a key factor determining its physiological performance and environmental thresholds, as different symbionts possess distinct thermal and photophysiological tolerances (Fautin & Buddemeier, 2004; Stat et al., 2013; Ivory et al., 2025). Moreover, flexible host-symbiont associations have been linked to broader ecological distributions, whereas specialized associations may restrict host range (Ziegler et al., 2015). Originally grouped under a single genus, *Symbiodinium*, molecular tools have since resolved this genus into multiple distinct lineages in the family Symbiodiniaceae (LaJeunesse et al., 2018; Pochon & LaJeunesse, 2021). Octocorals host symbionts historically classified into clades A–D and G, which are now recognized as distinct genera: *Symbiodinium*, *Breviolum*, *Cladocopium*, *Durusdinium*, and *Gerakladium*, respectively (Schubert, Brown & Rossi, 2016; LaJeunesse et al., 2018; Van de Water, Allemand & Ferrier-Pagès, 2018). Some symbiont genotypes have been consistently linked to increased thermal tolerance in coral hosts. For example, Stat & Gates (2011) demonstrated that corals associating with members of Clade D (*Durusdinium)* were more resistant to elevated sea surface temperatures. Similarly, many studies have supported the thermotolerance of *Durusdinium* species and the increased resistance to bleaching experienced by coral hosts harboring them (Baker et al., 2004; Ladner, Barshis & Palumbi, 2012; Cunning & Baker, 2013; Cunning et al., 2015; Cunning, Ritson-Williams & Gates, 2016; Silverstein, Cunning & Baker, 2017) Colonies hosting “Clade D” symbionts were abundant at inshore habitats with elevated turbidity (LaJeunesse et al., 2010b). Genotypes within *Symbiodinium* (Clade A) have also demonstrated elevated thermal tolerance (Winters et al., 2009; Krämer et al., 2012; Hume et al., 2015) and adaptations to high-light environments, including enhanced photoprotective capacity (Reynolds et al., 2008), secretion of mycosporine-like amino acids (MAAs) in culture (Banaszak, Lajeunesse & Trench, 2000), and physiological traits associated with high irradiance tolerance (Rowan et al., 1997).

The occurrence of distinct intraspecific coral morphotypes has been linked to varying environmental regimes, including depth, irradiance, and temperature gradients (Takabayashi & Hoegh-Guldberg, 1995; Frade et al., 2008a; Innis et al., 2018). A comparable pattern is observed in the distribution of morphotypes of the endemic octocoral *Sarcothelia edmondsoni* across Hawaiian reefs, where blue morphotypes consistently occupy high-energy, clear-water environments, while the brown morphotypes are more commonly found in calmer, turbid habitats. In addition to ecological differentiation, coral morphotypes often harbor distinct bacterial and Symbiodiniaceae assemblages, with composition varying significantly by depth- and color morph (Frade et al. 2008b, Frade et al., 2008; Shore-Maggio et al., 2015; Ziegler et al., 2015; Innis et al., 2018).

**Figure 1:**
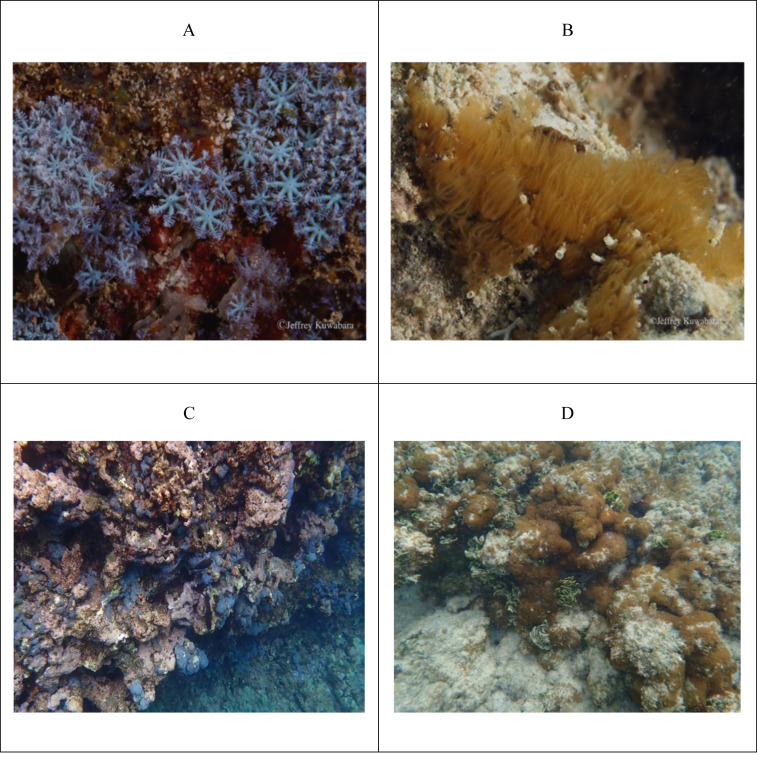
Morphotypes of *Sarcothelia edmondsoni*. (A, B) Close-up images of polyps from two distinct morphotypes: blue (A) and brown (B). (C, D) Representative images of the morphotypes *in situ*, with the blue morphotype (C) and brown morphotype (D) shown as relatively dominant on degraded reef habitats. Photo credit (A, B): Jeffrey Kuwabara.

The polyps of this endemic octocoral are soft-bodied and arise from a basal encrusting membrane, extending up to 10.0 mm in height (Verrill, 1928; Davis, 1977; Fig. 1). Coloration has been attributed to sclerite color and arrangement (Mercer & Singh, 1975), appearing blue in reflected light and amber in transmitted light (Verrill, 1928). Despite their morphological divergence, genetic markers examined to date (COI, *msh1*, and 28S rDNA) have not revealed any differences between the morphotypes (C. McFadden, pers. comm., 2021). In the only previous study on this species, its reproductive biology appears consistent across morphotypes (Davis, 1977).

While the distribution of *Sarcothelia edmondsoni* morphotypes across contrasting habitats suggests possible differences in physiological capacity and symbiont associations, the composition of its endosymbiotic communities remains poorly characterized. Early investigations reported Hawaiian colonies dominated by symbionts from Clade A (now *Symbiodinium*) (Mercer & Singh, 1975; Goulet, Simmons & Goulet, 2008). In contrast, LaJeunesse et al. (2004b) documented colonies at 10–20 m depth harboring a depth-specialist from Clade C (*Cladocopium,* specifically ITS2 type C15b). Additionally, a congeneric *Sarcothelia* species maintained in aquarium culture was found to harbor Clade D symbionts (*Durusdinium,* specifically type D4-5-9) (Parrin et al., 2016). Given the ecological divergence of *S. edmondsoni* morphotypes across reefs, we hypothesized that symbiont community composition would vary by morphotype. Although earlier studies reported associations between *S. edmondsoni* and *Symbiodinium*, we anticipated a greater prevalence of *Cladocopium* and *Durusdinium*—genera that are widely distributed among Hawaiian cnidarians and dominate Indo-Pacific coral symbioses (Knowlton & Rohwer, 2003; LaJeunesse et al., 2004b; Franklin et al., 2012). Characterizing the baseline symbiont assemblages of *S. edmondsoni* morphotypes provides critical insight into whether the resilience of this octocoral is mediated by its symbiotic associations.

Although soft corals are the second-most abundant macroinvertebrates on many Indo-Pacific reefs, their associations with dinoflagellate endosymbionts in the family Symbiodiniaceae remain significantly understudied compared to those of scleractinian corals (Schubert, Brown & Rossi, 2016; Jahajeeah, Bhoyroo & Ranghoo-Sanmukhiya, 2020). To address this gap, we used ITS2 profiling to examine the Symbiodiniaceae community structure associated with two visually distinct morphotypes of *Sarcothelia edmondsoni*—brown and blue—across three Hawaiian Islands. While ITS2 type profiles are often used to characterize symbiont diversity, they can represent either distinct species or intragenomic variants within a single lineage (Hume et al., 2019). Accordingly, we interpret ITS2 profiles as markers of symbiont diversity that may reflect population-level structure, intragenomic variation, or species boundaries. This approach enabled us to assess whether color morphotypes are associated with functionally distinct symbiont taxa—potentially reflecting differing physiological capacities—and to determine whether these associations vary geographically. Specifically, we asked: (1) Do brown and blue morphotypes of *S. edmondsoni* host distinct Symbiodiniaceae communities in terms of dominant taxa and relative abundance? (2) Are these community compositions consistent across islands, or do they exhibit spatial structuring? These questions are critical for understanding how host-symbiont partnerships contribute to coral ecological plasticity and stress resilience across variable reef environments.

## Materials & Methods

### Sample collection

**Figure 2:**
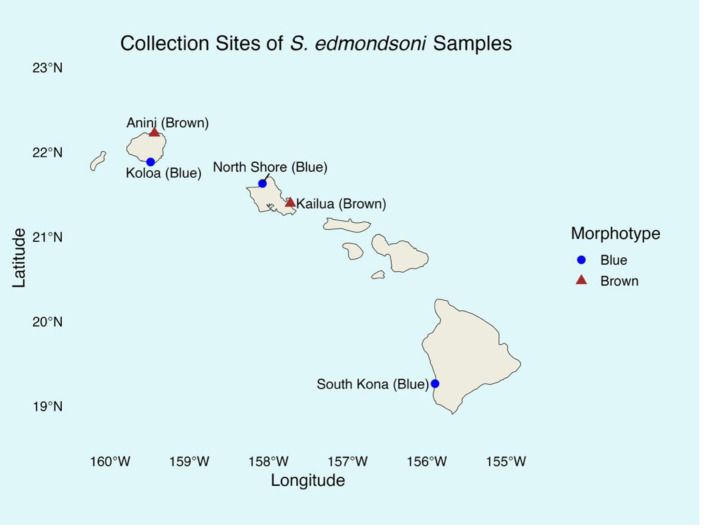
*Sarcothelia edmondsoni* collection sites with morphotype in parentheses. Note: *n* = 7 for all sites except South Kona (*n* = 5).

The study included both brown and blue morphotypes of *Sarcothelia edmondsoni* from three Hawaiian Islands (Fig. 2). Tissue samples from coral morphotypes were collected between March 2023 and January 2024 and stored in filtered seawater at −20 °C until DNA extraction in January 2025. All coral collections were authorized under the Division of Aquatic Resources Special Activities Permit SAP 2024-02. From Kauaʻi and Oʻahu, seven samples of each morphotype were analyzed. From Hawaiʻi Island, only five blue morphotype samples were collected due to time and funding constraints, but only four yielded successful sequencing data and were included in downstream analyses; no brown morphotypes from this site were found. Collection sites included: Leftovers, North Shore (blue, Oʻahu); Kailua Bay (brown, Oʻahu); Koloa (blue, Kauaʻi); Anini (brown, Kauaʻi); and South Kona (blue, Hawaiʻi Island). Although our sample sizes per morphotype and per island were modest, prior analyses have demonstrated that small sample sizes (≤5) can be sufficient to detect symbiont diversity in corals (Goulet, 2007), supporting the validity of our sampling approach.

### DNA extraction

Genomic DNA was extracted using the Qiagen DNeasy Blood & Tissue Kit per the manufacturer’s instructions. Approximately one polyp of host tissue was digested in ATL buffer with Proteinase K at 56 °C. Following lysis, DNA was purified on spin columns and eluted in AE buffer. DNA quality and quantity were assessed using NanoDrop and Qubit fluorometry.

### Library preparation and illumina MiSeq sequencing

Extracted DNA was submitted to the Advanced Studies in Genomics, Proteomics, and Bioinformatics (ASGPB) core facility at the University of Hawaiʻi at Mānoa. The ITS2 region was amplified using Symbiodiniaceae primers (Jacobs et al., 2021) with Illumina overhang adapters:

• Forward primer (454-ITSinfor2): 5′-TCGTCGGCAGCGTCAGATGTGTATAAGAG ACAGGAATTGCAGAACTCCGTG-3′
• Reverse primer (454-ITS2-reverse): 5′-GTCTCGTGGGCTCGGAGATGTGTATA AGAGACAGGGGATCCA TATGCTTAAGTTCAGCGGGT-3′

PCR amplification used Platinum Taq DNA Polymerase High Fidelity (Invitrogen). The first round included 35 cycles (95 °C for 30 s, 51 °C for 30 s, 68 °C for 30 s) and final extension at 68 °C for five minutes. Amplicons were checked on agarose gels and purified using Mag-Bind Total Pure NGS beads. A second PCR attached Nextera XT v2 index adapters with eight cycles and an annealing temperature of 55 °C. Libraries were purified, quantified with the PicoGreen dsDNA Assay Kit, normalized, and pooled. Final size distributions were evaluated on an Agilent Bioanalyzer High Sensitivity DNA chip. Sequencing was performed on an Illumina MiSeq with 2 × 300 bp paired-end reads.

### Symbiodiniaceae profiling *via* symportal

Next-generation sequencing data were submitted to SymPortal (Hume et al., 2019; https://symportal.org/), which characterizes Symbiodiniaceae community composition using a standardized framework that resolves defining intragenomic variants (DIVs) from ITS2 amplicon sequences. DIVs are specific ITS2 sequences that consistently co-occur across samples and serve as genetic “fingerprints” for identifying symbiont taxa.

In SymPortal terminology, a “type” refers to the most abundant ITS2 sequence (*e.g.*, A3), while an ITS2 type profile—also known as a DIV profile—consists of a characteristic set of co-occurring DIVs associated with that type (*e.g.*, A3-A3ad-A3fb). For each sample, SymPortal predicts these profiles based on both the presence and relative abundance of DIVs, yielding high-resolution taxonomic assignments that may correspond to species-level or lower resolution.

By referencing a global database of validated DIVs, SymPortal enables consistent ITS2 profiling across studies and improves resolution compared to traditional OTU-based approaches. Importantly, this method distinguishes intragenomic from intergenomic variation without requiring supplementary markers such as psbA^ncr^ (Hume et al., 2019). All downstream analyses of symbiont identity and diversity in this study are based on these SymPortal-defined ITS2 type profiles.

### Symbiont community ordination

To visualize community structure and compositional similarity among samples, Bray–Curtis dissimilarities calculated from normalized ITS2 type profile abundances were used for ordination analyses. These were conducted using both Principal Coordinates Analysis (PCoA) and Non-metric Multidimensional Scaling (NMDS). These visualizations provided insight into the multivariate clustering of samples by morphotype, location, and their interaction.

### ITS2 DIV-based phylogenetic dissimilarity

To assess the diversity, structure, and phylogenetic relatedness of Symbiodiniaceae communities across *Sarcothelia edmondsoni* morphotypes and geographic locations, we constructed a neighbor-joining (NJ) tree based on uncorrected pairwise p-distances from aligned ITS2 sequences. Sequences were imported into R using read.dna() from the ape package (Paradis et al., 2002), and pairwise genetic distances were calculated using dist.dna() with the “raw” model (proportion of nucleotide differences). An unrooted neighbor-joining tree was generated with nj() and visualized with plot.phylo(), with a scale bar denoting uncorrected p-distance (*i.e.,* proportion of nucleotide differences), scaled to approximate base pair differences based on alignment length. Tip labels were renamed to reflect ITS2 DIV identifiers for clarity.

Pairwise genetic distances were calculated and visualized as a heatmap using the ComplexHeatmap package (Gu, Eils & Schlesner, 2016). Hierarchical clustering was applied to both rows and columns using Euclidean distance and complete linkage. Cell values were rounded to three decimal places and overlaid in each heatmap cell. A white-to-blue color ramp and dynamic label contrast enhanced readability.

### UniFrac-based phylogenetic dissimilarity

Weighted UniFrac distances, which account for both phylogenetic divergence and relative abundance, were generated in the SymPortal analysis. The resulting distance matrix was processed in R v4.4.0 (R Core Team, 2025), where we constructed a NJ tree using the nj() function from the ape. Tree topologies were visualized in various layouts using plot.phylo Topological fidelity was assessed by comparing cophenetic distances to the original UniFrac matrix *via* Pearson correlation (R^2^).

The same matrix was visualized as a heatmap using ComplexHeatmap (Gu, Eils & Schlesner, 2016). with hierarchical clustering applied to rows and columns.

### Geospatial context

Sample collection sites were visualized using the ggplot2 (Wickham et al., 2007), sf (Pebesma, 2016), and ggrepel (Slowikowski, 2016) packages in R to produce a georeferenced map with annotated coordinates, contextualizing spatial variation in symbiont community structure.

### Statistical analysis of community composition

To formally test differences in Symbiodiniaceae community composition, we applied PERMANOVA using the adonis2() function from the vegan package (Oksanen et al., 2001). Analyses were performed on both Bray-Curtis and weighted UniFrac distance matrices. Pairwise comparisons among morphotypes and island combinations were conducted using pairwise.adonis() (Martinez Arbizu, 2020) function to identify significant group-level contrasts, enabling direct testing of group combinations (*e.g.*, island × morphotype).

## Results

### Sequences

High-throughput ITS2 sequencing yielded 705,966 Symbiodiniaceae sequences across 32 *Sarcothelia edmondsoni* samples. After quality control and Minimum Entropy Decomposition (MED) in SymPortal, we identified 145 unique ITS2 sequence variants and 14 ITS2 profiles (Table 1). Based on relative sequence abundances—calculated as the proportion of ITS2 sequences assigned to each DIV within a sample and summed across all samples—the community was overwhelmingly dominated by the genus *Symbiodinium* (99.1% of sequences). The dominant ITS2 sequence was A3 (*Symbiodinium tridacnidorum*), comprising 88.9% of total sequences. The 14 ITS2 profiles were distributed across four Symbiodiniaceae genera: *Symbiodinium*, *Breviolum*, *Cladocopium*, and *Fugacium* (Table 2). While sequences for *Breviolum* and *Fugacium* were detected, they were removed from subsequent analyses as they did not co-occur to form any ITS2 type profiles and only occurred in one sample each at low relative abundance, suggesting they likely do not regularly associate with *S. edmondsoni*. After removing *Breviolum* and *Fugacium* profiles due to their low abundance and lack of co-occurrence, the number of ITS2 profiles retained for downstream analysis was reduced to 12.

**Table 1:** Summary of ITS2 profiles found in each *Sarcothelia edmondsoni* morphotype on each island sampled. The top three profiles (relative abundances exceeding 1%) are detailed. The numbers in parentheses indicate the number of profiles found exclusively in that island or morphotype.

Location/Morph	ITS2 profiles	Top 3 profiles (in order of abundance)
**Oʻahu**	7 (6)	A3-A3ad-A3fb, A3/A3ah, A3/A3g
Oʻahu blue	5 (5)	A3-A3ad-A3fb, A3bt
Oʻahu brown	2 (1)	A3/A3ah, A3/A3g
**Kauaʻi**	4 (3)	A3-A3u, A3/A3ez-A3ey, A4ac/A3-A4eo
Kauaʻi blue	3 (3)	A3/A3ez-A3ey, A4ac/A3-A4eo, A3ez
Kauaʻi brown	1 (0)	A3-A3u
**Hawaiʻi Island**	3 (1)	A3-A3fa, A3/A3g, A3-A3u
**Blue**	11 (9)	A3-A3ad-A3fb, A3/A3ez-A3ey, A3-A3fa
**Brown**	3 (1)	A3-A3u, A3/A3ah, A3/A3g
**Total**	12	A3-A3u, A3-A3ad-A3fb, A3/A3ah

**Table 2:** Symbiodiniaceae community composition (genus level) by *Sarcothelia edmondsoni* morph and location. Values represent the percentage of each genus present.

Morphotype	Location	*Symbiodinium* (%)	*Cladocopium* (%)
Blue	Hawaiʻi Island	100.0	0.0
Blue	Kauaʻi	100.0	0.0
Blue	Oʻahu	99.5	0.5
Brown	Oʻahu	100.0	0.0
Brown	Kauaʻi	100.0	0.0

The top five non-A3 Clade A ITS2 sequence variants (by relative abundance) included A4ac (3.6%), A4eo (0.40%), 5517405_A (0.37%), 1463150_A (0.26%), and s64769_A (0.26%). In contrast, non-Clade A sequences—those belonging to other Symbiodiniaceae genera—comprised only 0.90% of the total dataset, indicating that *S. edmondsoni* colonies in this study are overwhelmingly dominated by Clade A taxa, with A3-type lineages representing the core symbiotic community.

**Figure 3:**
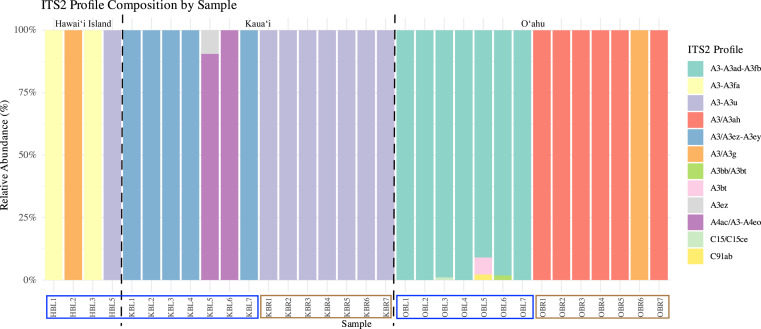
Relative ITS2 profile composition across individual samples of *Sarcothelia edmondsoni* morphotypes collected from Hawaiʻi Island, Kauaʻi, and Oʻahu. Each bar represents an individual coral sample, with segments indicating the relative abundance of distinct ITS2 profiles. To aid interpretation, a dotted black line separates islands, and sample names along the *x*-axis have been outlined according to morphotype: blue outlines indicate blue morphs, and brown outlines indicate brown morphs.

Symbiont community diversity varied markedly by morphotype and island. Blue morphotypes hosted substantially higher diversity, comprising 129 unique ITS2 sequence variants and 12 distinct ITS2 profiles, whereas brown morphotypes harbored only 30 unique ITS2 variants and three profiles (Fig. 3). This pattern was most pronounced in Oʻahu samples: blue morphotypes from Oʻahu exhibited the greatest within-group diversity with 51 unique ITS2 variants and five ITS2 profiles, while brown morphotypes from Kauaʻi represented the least variable group, with just 18 ITS2 variants and 1 ITS2 profile (Table 1, Fig. 3).

**Figure 4:**
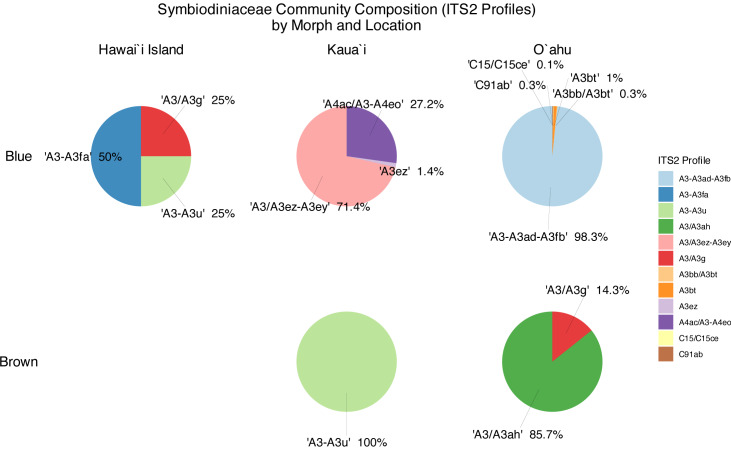
Relative abundance (>0.1%) of ITS2 profiles in blue (top) and brown (bottom) morphotypes of *Sarcothelia edmondsoni* collected from Hawaiʻi Island, Kauaʻi, and Oʻahu.

Across locations, both Oʻahu and Kauaʻi hosted 59 unique ITS2 sequence variants. However, community overlap was low: only 10 ITS2 DIVs were shared between the two islands, and no ITS2 profile was common across all morphotype–location combinations (Fig. 4). Similarly, no single ITS2 profile was observed in both Oʻahu and Kauaʻi samples. Nonetheless, inter-island connections were evident. For example, the A3/A3g ITS2 profile was shared between Hawaiʻi Island and Oʻahu samples, while A3-A3u appeared in both Hawaiʻi Island and Kauaʻi. At the sequence level, A3 was ubiquitous—detected across all samples—whereas A3u and A3bt were found in all groups, underscoring their widespread distribution.

### Symbiont community composition

To explore how symbiont communities varied across morphotypes and islands, we employed two complementary ordination strategies based on different ecological distance metrics: Bray-Curtis and UniFrac. These approaches capture distinct aspects of community structure—Bray-Curtis emphasizes compositional dissimilarity based on ITS2 type abundances, while UniFrac integrates phylogenetic relatedness alongside sequence abundance, offering a more evolutionarily-informed perspective.

**Figure 5:**
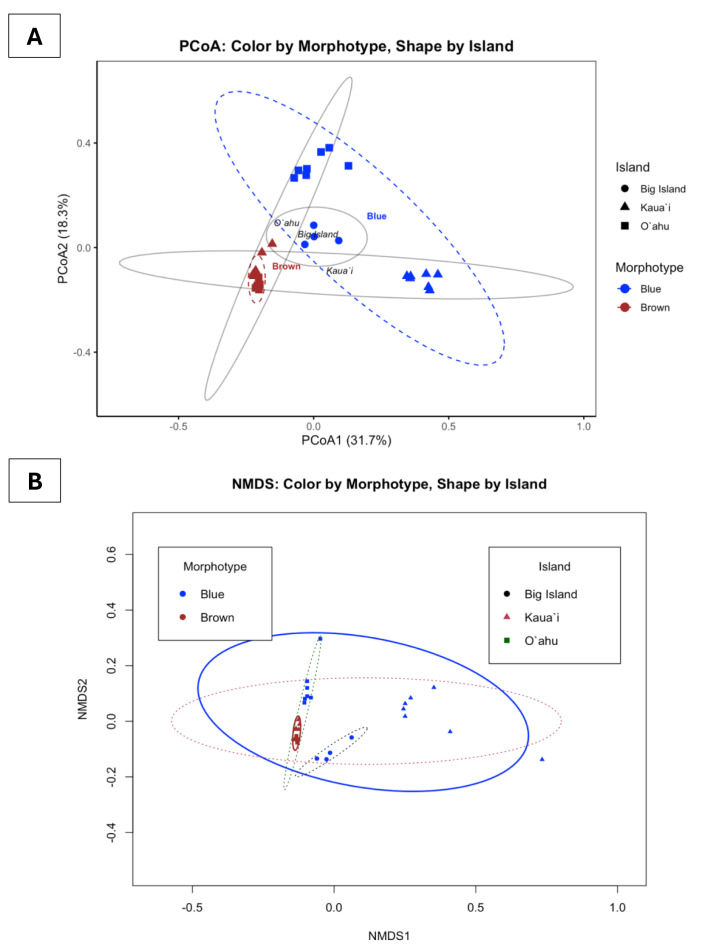
Principal coordinates analysis (PCoA) and non-metric multidimensional scaling (NMDS) of ITS2 profiles found in *Sarcothelia edmondsoni* morphotypes across three Hawaiian Islands. (A) PCoA with points colored by *Sarcothelia edmondsoni* morphotype (brown = brown, blue = blue) and shaped by island (circle = Hawaiʻi Island, triangle = Kauaʻi, square = Oʻahu). Ellipses represent one standard deviation around groups. PCoA1 and PCoA2 collectively explain 50% of the total variation in community composition. (B) NMDS with points colored by morphotype and shaped by island. Color of ellipses also corresponds to island or morphotype (black/circle = Hawaiʻi Island, pink/triangle = Kauaʻi, green/square = Oʻahu). Ellipses represent one standard deviation around each group. NMDS stress = 0.08, indicating an excellent 2D fit.

Bray-Curtis-based multivariate analyses revealed strong structuring of Symbiodiniaceae communities. PCoA and NMDS ordinations revealed pronounced clustering of samples by both morphotype and island (Fig. 5). Blue and brown morphotypes formed largely discrete clusters, while samples from Kauaʻi, Oʻahu, and Hawaiʻi Island showed geographically differentiated patterns. These groupings were visually reinforced by the statistical ellipses, which showed well-defined group dispersions with limited overlap, suggesting non-random community partitioning. The NMDS ordination, characterized by a low stress value (0.0858), reliably captured the community structure, while the first two PCoA axes together explained approximately 50% of the variation.

To statistically test these visual patterns, we conducted a factorial permutational multivariate analysis of variance (PERMANOVA) using the *adonis2* function on Bray–Curtis distance matrices (square-root transformed). The full model, which included Morphotype, Location, and their interaction, was highly significant (F(4, 27) = 7.2961, *R*^2^ = 0.5194, *p* = 0.001), explaining over 51% of the total variance in symbiont community composition. When tested independently, both Morphotype (F(1,30) = 7.489, *R*^2^ = 0.199, *p* = 0.001) and Location (F(2,29) = 3.813, *R*^2^ = 0.208, *p* = 0.001) showed significant effects, each accounting for roughly 20% of the observed variation.

The interaction term in the full model suggests that the effect of morphotype on community composition may vary by location, or conversely, that geographic patterns are dependent on morphotype identity. Follow-up pairwise PERMANOVAs confirmed significant differences in community structure between all island pairs—Kauaʻi *vs.* Oʻahu (F(3,24) = 9.4195, *R*^2^ = 0.5407, *p* = 0.001), Kauaʻi *vs.* Hawaiʻi Island (F(2,15) = 9.3067, *R*^2^ = 0.5537, *p* = 0.001), and O’ahu *vs.* Hawaiʻi Island (F(2,15) = 4.2857, *R*^2^ = 0.3636, *p* = 0.001). These findings provide robust statistical support for spatially and morphologically structured symbiont assemblages.

Collectively, the congruence between NMDS and PCoA, along with strong PERMANOVA results, supports the conclusion that *S. edmondsoni* morphotypes and their geographic distributions are associated with ecologically structured and potentially adaptive symbiont communities. These patterns may reflect a combination of host–symbiont specificity, environmental adaptation, or localized selective pressures acting on symbiont assemblages.

**Figure 6:**
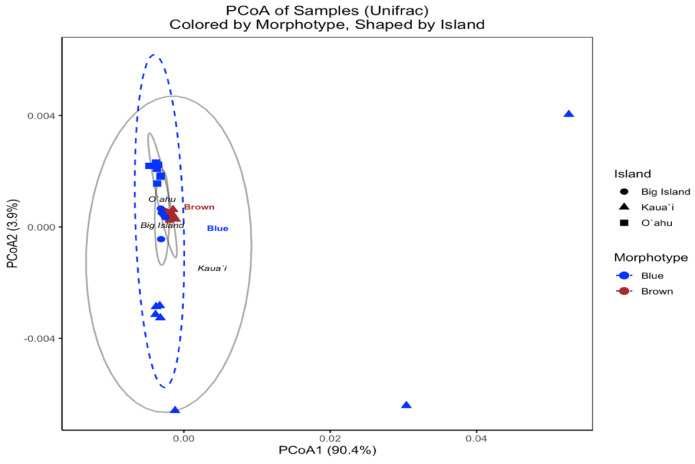
UniFrac principal coordinates analysis (PCoA) of *Symbiodinium* ITS2 type profiles from different *Sarcothelia edmondsoni* morphotypes collected across three Hawaiian Islands. The analysis is based on UniFrac distances. Points are colored by morphotype (blue or brown) and shaped by island of origin (circle = Hawaiʻi Island, triangle = Kauaʻi, square = Oʻahu). Ellipses represent the multivariate dispersion of samples within morphotype groups (dashed) and island groups (solid gray). Axis labels reflect the percentage of variation explained by the first two principal coordinates.

In contrast, the UniFrac-based PCoA—calculated from square-root-transformed phylogenetic distances—provided insight into the evolutionary relatedness of symbiont communities across samples. The first two principal coordinates captured 90.0% and 3.8% of the variation, respectively, with samples broadly clustering by island (Fig. 6). However, brown and blue morphotypes were intermixed within islands, and PERMANOVA results indicated no statistically significant differences by Island (F(2, 29) = 1.555, *R*^2^ = 0.097, *p* = 0.13) or Morphotype (F(1, 30) = 1.052, *R*^2^ = 0.034, *p* = 0.473). These findings suggest that although Bray–Curtis analyses revealed strong structuring in community composition, the dominant symbiont profiles across morphotypes and islands are not phylogenetically divergent.

## Discussion

The symbiont community composition of *Sarcothelia edmondsoni* revealed strong structuring by host morphotype. These findings are important because they reveal that host morphology and geography jointly structure symbiont community composition, offering insight into coral-symbiont specificity, ecological adaptation, and potential resilience under environmental stress. The strikingly higher diversity observed in blue morphotypes—hosting over four times the number of ITS2 DIVs and profiles compared to brown morphs—may indicate greater symbiotic flexibility or broader ecological tolerance. Such flexibility could confer adaptive advantages under variable environmental regimes, potentially helping explain their observed association with higher-energy reef environments or in upper-mesophotic depths. In contrast, the comparatively depauperate symbiont communities in brown morphotypes suggest more constrained partnerships, possibly indicative of narrower ecological specialization.

The lack of shared ITS2 profiles across the morphotype-location groups underscores the specificity of these associations, reinforcing the idea that host morphotype and geography act as strong filters on symbiont acquisition or maintenance. While some ITS2 DIVs (*e.g.*, A3, A3u, A3bt) were ubiquitous across samples, they consistently co-occurred with ITS2 DIVs that were specific to morphotypes or locations. For instance, blue morphs from Oʻahu exhibited the highest number of distinct ITS2 profiles, whereas brown morphs from Kauaʻi were associated with only a single profile. This contrast points to clear differences in symbiont diversity linked to host morphotype and also raises the possibility that some symbiont profiles may be unique to certain islands—potentially indicating localized endemism.

The lack of overlap in ITS2 profiles between Oʻahu and Kauaʻi also points to potential regional structuring of Symbiodiniaceae communities. This could reflect limited symbiont dispersal, environmental selection, or historical divergence in host-symbiont coevolution. While our per-island and per-morphotype sample sizes were limited—and additional sampling may reveal greater overlap—the observed differences suggest meaningful patterns that merit further investigation. Such spatial and morphotype-specific differences in symbiont composition have important implications for the thermal tolerance and ecological resilience of *S. edmondsoni*, as symbiont identity may influence host performance under stress—though additional research is needed to test this hypothesis.

The predominant repeatable ITS2 profiles retrieved from *S. edmondsoni* were largely dominated by *Symbiodinium* “A3” variants, which likely correspond to subtypes or subspecies within *S. tridacnidorum,* a widely distributed Indo-Pacific symbiont originally described from giant clams and Pacific upside-down jellyfish, *Cassiopea* (Lee et al., 2015). In this dataset, 88.9% of total sequences across all samples may be considered likely intragenomic variants of the dominant A3 symbiont, underscoring a highly conserved symbiotic community structure. Sequences from *Symbiodinium,* in total, comprised 99.1% of the ITS2 sequence abundance (Table 2), suggesting that meaningful biological interpretation should focus on the dominant community signals rather than low-abundance variation. Due to the multicopy nature of the ITS2 region and the amplification sensitivity of high-throughput platforms such as Illumina MiSeq, previous studies have emphasized that small sequence differences must be interpreted with caution, as they may reflect rare intragenomic variants or PCR artifacts rather than distinct taxa (Thornhill, Lajeunesse & Santos, 2007; Sampayo, Dove & Lajeunesse, 2009; LaJeunesse & Thornhill, 2011; Davies et al., 2023).

**Figure 7:**
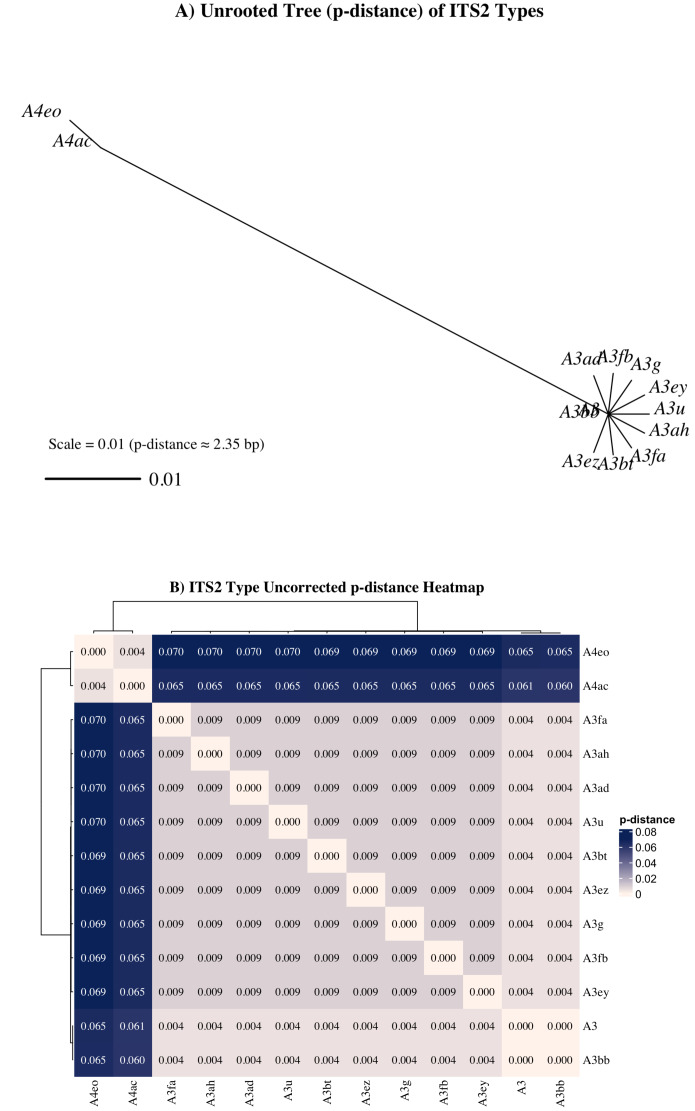
Unrooted neighbor-joining (NJ) tree and heatmap from aligned ITS2 sequences representing distinct *Symbiodinium* types found in *Sarcothelia edmondsoni* morphotypes. (A) NJ tree was constructed using pairwise p-distances calculated from aligned ITS2 sequences. The scale bar represents a p-distance of 0.01, corresponding approximately to a 1% sequence divergence (∼2.35 bp for this alignment). (B) Heatmap of uncorrected p-distances among the same ITS2 types, calculated from the aligned sequence dataset. Hierarchical clustering was applied to both rows and columns.

Nonetheless, a subset of sequences—particularly those associated with the A4ac/A3-A4eo profile—may represent true taxonomic divergence. A4 sequences exhibited a minimum uncorrected genetic distance (*p* = 0.061) from A3 sequences (Fig. 7), exceeding the species-level divergence threshold (*p* ≥ 0.04) proposed by Litaker et al. (2007) for free-living dinoflagellates. Additionally, UniFrac distances between A4ac/A3-A4eo and the dominant A3-type profiles also met or exceeded this threshold, reinforcing the potential distinctiveness of this profile (Fig. S1). Although A4ac/A3-A4eo may correspond to *Symbiodinium linucheae*, the A4 species originally described from Caribbean hosts (Trench & Thinh, 1995), it may alternatively represent a previously undescribed Indo-Pacific *Symbiodinium* species. However, given that ITS2 amplifications often retrieve multiple co-occurring sequence variants—even from genetically uniform symbiont populations—this apparent divergence must be cautiously interpreted. LaJeunesse & Thornhill (2011) demonstrated that ancestral ITS2 sequence variants can be retained at low copy number within the rDNA arrays of derived lineages and co-amplified during PCR, complicating assessments of true symbiont diversity. Thus, it remains highly plausible that the A4-type sequences observed here represent residual ancestral rDNA variants within *Symbiodinium* “A3” rather than evidence of active co-occurrence with A4 taxa. Given that most cnidarians tend to host a single symbiont species or combinations from phylogenetically distinct genera (LaJeunesse & Thornhill, 2011; LaJeunesse et al., 2022; Davies et al., 2023), the observed A4 sequences are more likely intragenomic remnants than true co-residents. Further validation with additional molecular markers (*e.g.*, psbA^*n*^, LSU) would be required to determine the taxonomic status of A4ac/A3-A4eo.

Although Bray-Curtis-based analyses indicated significant structuring of symbiont communities by morphotype and island, the lack of phylogenetic separation in the UniFrac-based PERMANOVA suggests that these differences largely occur within a closely related group of symbionts. This supports the interpretation that the observed variation likely reflects fine-scale ecological differentiation or intragenomic diversity within a single species or species complex, rather than distinct symbiont lineages (LaJeunesse & Thornhill, 2011; Thornhill et al., 2017; Davies et al., 2023).

Octocorals are known to harbor symbionts in five of the currently described Symbiodiniaceae genera: *Symbiodinium, Breviolum, Cladocopium, Durusdinium,* and *Gerakladium* (clades A-D, G; Jahajeeah, Bhoyroo & Ranghoo-Sanmukhiya, 2020). Consequently, the background population of *Fugacium* “F5.1” is likely transient, commonly found coating coral tissue surfaces (LaJeunesse et al., 2018; Fujise et al., 2021; Ng et al., 2024). Similarly, given its exceptionally low abundance in this study—and considering that *Breviolum* is the dominant symbiont on Caribbean reefs (LaJeunesse, 2002; Finney et al., 2010; LaJeunesse et al., 2018)—the presence of *Breviolum* “B1aw” is likely indicative of either contamination or a transitory population. This interpretation is further supported by evidence that *B. minutum* is the homologous symbiont in the glass anemone *Exaiptasia diaphana* (Thornhill et al., 2013; Dungan et al., 2020; Tortorelli et al., 2020), a widespread species commonly found on Hawaiian reefs and tidepools. Previous research has detected *Cladocopium* “C15” symbionts in *S. edmondsoni* samples (Goulet, Simmons & Goulet, 2008).

The consistent association of distinct ITS2 profiles with specific morphotypes and reef locations suggests the possibility of *Symbiodinium* ecotypes within the A3 radiation. While the dominant presence of A3 across samples indicates a conserved symbiont lineage, variation in co-occurring intragenomic variants and their structured distribution hint at ecologically differentiated strains. Notably, physiological differences among strains within the same species have been experimentally demonstrated. For instance, Díaz-Almeyda et al. (2017) found that thermotolerance varied significantly both between species within *Symbiodinium* and among strains of the same species, underscoring the potential for fine-scale functional variation within a single species. These patterns may represent adaptation to specific host morphotypes and localized environmental microhabitats, supporting the notion of functional specialization within a taxonomic lineage. Such specialization could reflect ecological filtering along habitat gradients or host trait selection, indicating potential for symbiont ecotypes tailored to distinct environmental or biological contexts. This study is consistent with previous studies that have found that zooxanthellate corals typically associate with one dominant symbiont genotype (LaJeunesse & Thornhill, 2011; Thornhill et al., 2017) and highlights how morphotype-specific associations may offer insight into the functional ecology and resilience of this octocoral.

Reynolds et al. (2008) revealed significant physiological differences in photoprotective capacity among endosymbiont genera. Members of *Symbiodinium* (Clade A)—notably *S. tridacnidorum* (A3) and A4a—demonstrated specialized adaptations to high-light environments, including photosystem II (PSII)-independent cyclic electron transport (CET), chlororespiration (in which oxygen acts as an alternative electron acceptor), and light-induced dissociation of both peridinin–chlorophyll–protein (PCP) and intrinsic light-harvesting complexes (LHCs) from PSII. These mechanisms collectively sustain ATP production and mitigate photodamage during stress, thereby preserving photosynthetic function under conditions that may otherwise promote bleaching. In contrast, symbionts from *Breviolum* (Clade B) and *Cladocopium* (Clade C), typically found in deeper-water corals, lacked CET activity, displayed weak chlororespiration, and retained coupled antenna systems under high light.

Similarly, early studies showed that cultured *Symbiodinium* symbionts, including *S. tridacnidorum*, synthesized mycosporine-like amino acids (MAAs) in response to UV radiation, enhancing their photoprotective capacity (Banaszak, Lajeunesse & Trench, 2000; LaJeunesse, 2001). It has been suggested that this likely contributes to the prevalence of *Symbiodinium* in corals inhabiting shallow reefs in high-irradiance environments (LaJeunesse, 2002).

These patterns imply that *S. tridacnidorum* symbionts have evolved a suite of constitutive photoprotective responses suited to high-irradiance environments, potentially conferring greater resilience under conditions linked to coral bleaching. Despite these photoprotective traits, Cumbo, Van Oppen & Baird (2018) characterized A3 symbionts as “heat-sensitive” as they exhibit heightened sensitivity to elevated temperatures, rapidly losing photosynthetic capacity at 34 °C. Similarly, Díaz-Almeyda et al. (2017) found *S. tridacnidorum* to be one of the most thermally sensitive species within the genus, with both tested strains exhibiting pronounced declines in cell density and photosynthetic performance at 32 °C. This contrast suggests that while *S. tridacnidorum* may be well-adapted to light stress, its thermal tolerance may be more limited than previously assumed.

The trophic plasticity of species like *S. edmondsoni* enables flexible nutrient acquisition, potentially buffering physiological stress. While *S. tridacnidorum* may confer certain benefits under high irradiance (*e.g.*, increased MAAs), it remains unclear to what extent the symbionts influence the Hawaiian soft coral’s ecological threshold or physiology. Octocorals are more mixotrophic than scleractinians, demonstrating greater dependency on heterotrophy compared to scleractinians (Fabricius & Klumpp, 1995). This has been linked to greater stress resistance in soft corals as symbiont loss (*i.e.,* coral bleaching) may not ultimately alter the energetic input of the octocoral (Anthony & Fabricius, 2000; Grottoli, Rodrigues & Palardy, 2006; Ferrier-Pagès, Sauzéat & Balter, 2018). It has been suggested that octocorals exhibit lower symbiont diversity than scleractinians due to greater reliance on heterotrophy and subsequent reduced dependence on symbiont flexibility (Baker & Romanski, 2007).

## Future research directions

Further research is needed to clarify the mechanisms shaping symbiont community structure in *Sarcothelia edmondsoni*, particularly in relation to its reproductive strategy and environmental distribution. Previous research has suggested host-symbiont specificity is common for vertically transmitting hosts (LaJeunesse et al., 2004a). While vertical transmission of symbionts is observed in approximately 60% of brooding octocorals (Schubert, Brown & Rossi, 2016), *S. edmondsoni* is an external brooder with documented external fertilization (Davis, 1977). This reproductive mode increases the possibility of horizontal symbiont acquisition from the environment. Clarifying whether this species transmits symbionts vertically or horizontally will be critical to understanding the strong symbiont specificity observed across morphotypes and islands.

Although this study focused on shallow-water colonies, it is noteworthy that the blue morphotype of *S. edmondsoni* has been documented at depths up to 55 m (S. Rowley, pers. comm., 2022). It remains unknown which symbiont species are associated with these deep colonies, or whether symbiosis plays an active role in facilitating their extended depth range. There is also the possibility that these upper-mesophotic colonies are facultatively symbiotic—or even azooxanthellate—mirroring patterns seen in the Mediterranean gorgonian *Eunicella singularis*, which hosts symbionts in shallow water (up to 30 m) but becomes symbiont-free at 40–60 m (Gori et al., 2012). This possibility is consistent with broader trends in octocoral ecology, where approximately 75% of described species occur at depths >50 m and are predominantly nonsymbiotic (Schubert, Brown & Rossi, 2016).

Previous studies have identified depth as a significant driver of symbiont community composition in corals. For example, *Montipora* morphotypes have shown a shift from *Durusdinium* in shallow water (<two m) to *Cladocopium* at greater depths (Innis et al., 2018). Moreover, LaJeunesse et al. (2004b) reported associations of *S. edmondsoni* with depth-specialist C15b at 10–20 m on Oʻahu. Such patterns support the hypothesis that deeper *S. edmondsoni* populations—particularly blue morphotypes—may harbor distinct symbionts adapted to low-light conditions. Furthermore, De Souza et al. (2022) demonstrated that depth and temperature variability can account for a significant portion (∼20%) of variation in symbiont community structure, underscoring the value of sampling across environmental gradients. Future studies should prioritize characterizing symbiont composition across depth, light, and temperature regimes to evaluate whether morphotype-specific or ecotypic associations influence the distribution and resilience of *S. edmondsoni*—a fast-colonizing endemic octocoral that dominates many of Hawaii’s degraded reefs. Research that includes site-specific environmental data will be essential for explicitly linking habitat conditions to symbiont community structure. Such insights are crucial for informing reef management and understanding the ecological role of this species in the context of ongoing environmental change.

## Conclusions

These findings align with previous research indicating that interactions between host specificity and local environmental conditions influence symbiont community composition (Coffroth & Santos, 2005). They also highlight the intricate and highly specific nature of symbiotic relationships in *Sarcothelia edmondsoni*, where both host morphotype and geographic variation distinctly structure associations with *Symbiodinium*, particularly *S. tridacnidorum*. As the most prevalent *Symbiodinium* species in the Indo-Pacific (Lee et al., 2015), *S. tridacnidorum* may influence host photophysiology and environmental range, especially under high-irradiance conditions.

This study also highlights the ecological relevance of octocorals as model systems for understanding symbiotic plasticity—an area that remains critically understudied compared to their scleractinian counterparts (Schubert, Brown & Rossi, 2016). With their trophic flexibility and broader depth distribution, species like *S. edmondsoni* offer valuable insight into how symbiotic associations can be structured, sustained, and potentially adapted under environmental stress. The observed morphotype-linked and site-specific symbiont structuring, alongside evidence of conserved but potentially ecotypically differentiated *Symbiodinium* lineages, underscores the importance of considering both host traits and local context in evaluating coral–symbiont dynamics. As climate change continues to intensify stress on coral reef ecosystems, deepening our understanding of these partnerships—particularly in overlooked taxa like octocorals—will be essential for predicting and sustaining coral resilience across diverse reef habitats.

## Supplemental Information

10.7717/peerj.20549/supp-1 Supplemental Information 1 Phylogenetic tree and heatmap of UniFrac distances (A) Neighbor-Joining (NJ) tree constructed from weighted UniFrac distances among Symbiodinium ITS2 type profiles. Each tip represents a distinct community profile (DIV), as identified by SymPortal. Weighted UniFrac distances incorporate both sequence divergence and relative abundance of ITS2 variants. The tree is shown in an unrooted layout, and branch lengths are scaled to reflect UniFrac-derived evolutionary distances. Tree fit (R^2^) = 1.000 (B) Heatmap of the same weighted UniFrac distance matrix, clustered hierarchically to visualize pairwise dissimilarities among ITS2 type profiles. A blue gradient color scheme reflects increasing distance. Together, tree and heatmap illustrate community-level beta diversity among coral-associated symbiont populations.

## Additional Information and Declarations

### Field Study Permissions

The following information was supplied relating to field study approvals (i.e., approving body and any reference numbers): Sample collection was conducted under a Special Activity Permit (SAP 2024-02) issued by the Hawaiʻi Department of Land and Natural Resources (DLNR), Division of Aquatic Resources (DAR).

### DNA Deposition

The following information was supplied regarding the deposition of DNA sequences: The Symbiodiniaceae ITS2 sequences described here are uploaded to SymPortal and will additionally be accessible through GenBank accession numbers PX124637–PX124778.

### Data Availability

The following information was supplied regarding data availability: The data is available at GitHub and Zenodo. https://github.com/ecabell/Sarcothelia_ITS2
ecabell. (2025). ecabell/Sarcothelia_ITS2: Release (v1.2). Zenodo. https://doi.org/10.5281/zenodo.17654129. The Symbiodiniaceae ITS2 sequences are available at SymPortal:
https://symportal.org/data_explorer/?Study=202506_cabeler1_Hawaii_SarcoEd.
