# Supplementary material for: Spatial and host-specific structuring in symbiont community composition of an endemic Hawaiian octocoral, Sarcothelia edmondsoni (Verrill 1928)
Source: PeerJ. 2026 Jan 13;14:e20549. doi: 10.7717/peerj.20549 (PMC12810398; doi:10.7717/peerj.20549)
Supplement: Supplemental Information 1 — (A) Neighbor-Joining (NJ) tree constructed from weighted UniFrac distances among Symbiodinium ITS2 type profiles. Each tip represents a distinct community profile (DIV), as identified by SymPortal. Weighted UniFrac distances incorporate both sequence divergence and relative abundance of ITS2 variants. The tree is shown in an unrooted layout, and branch lengths are scaled to reflect UniFrac-derived evolutionary distances. Tree fit (R2) = 1.000 (B) Heatmap of the same weighted UniFrac distance matrix, clustered hierarchically to visualize pairwise dissimilarities among ITS2 type profiles. A blue gradient color scheme reflects increasing distance. Together, tree and heatmap illustrate community-level beta diversity among coral-associated symbiont populations. [file peerj-14-20549-s001.pdf]

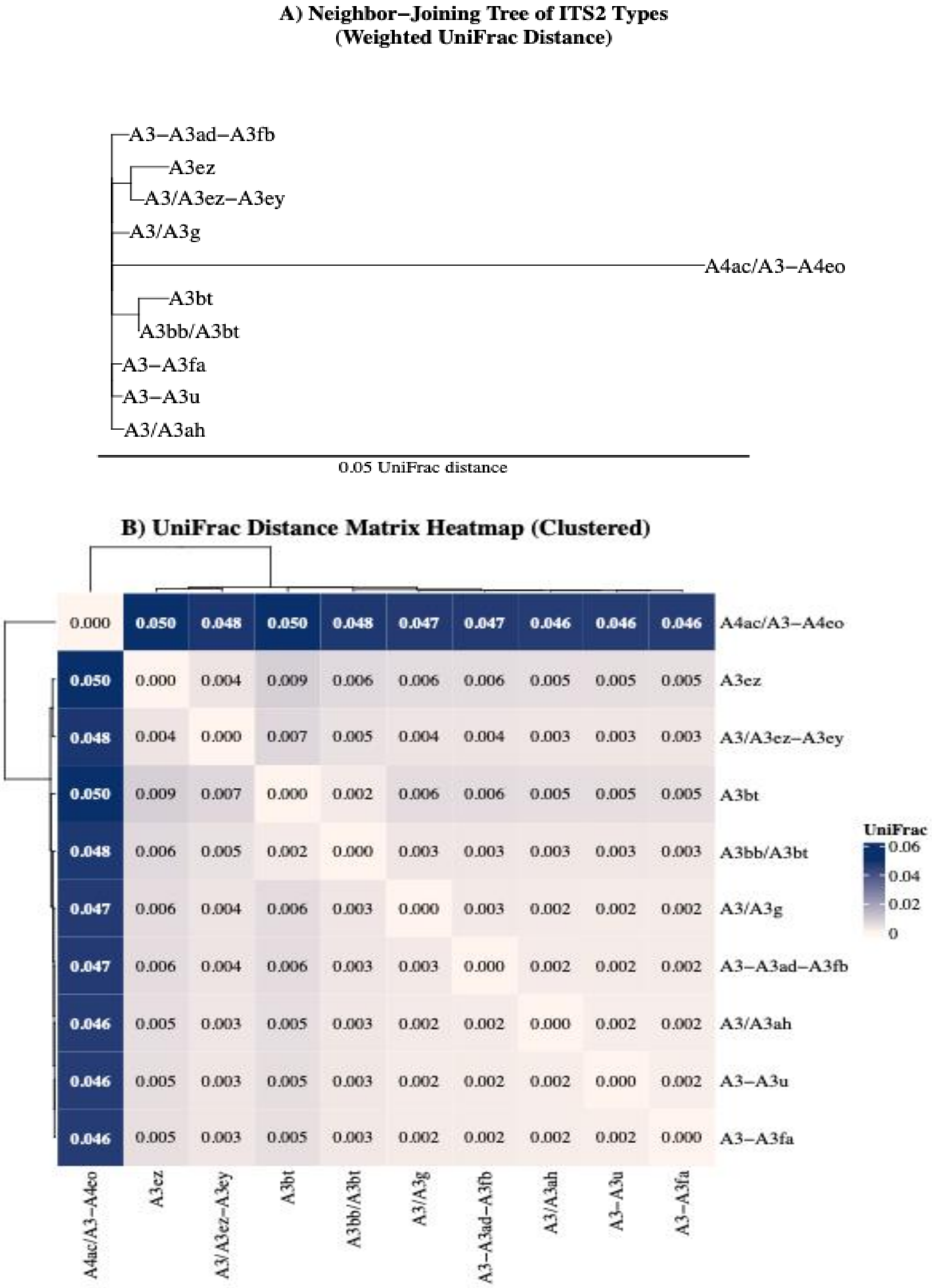
A) Neighbor−Joining Tree of ITS2 Types
(Weighted UniFrac Distance)
A3−A3ad−A3fb
A3ez
A3/A3ez−A3ey
A3/A3g
A4ac/A3−A4eo
A3bt
A3bb/A3bt
A3−A3fa
A3−A3u
A3/A3ah
0.05 UniFrac distance
B) UniFrac Distance Matrix Heatmap (Clustered)
0.000 0.050 0.048 0.050 0.048 0.047 0.047 0.046 0.046 0.046 A4ac/A3−A4eo
0.050 0.000 0.004 0.009 0.006 0.006 0.006 0.005 0.005 0.005 A3ez
0.048 0.004 0.000 0.007 0.005 0.004 0.004 0.003 0.003 0.003 A3/A3ez−A3ey
0.050 0.009 0.007 0.000 0.002 0.006 0.006 0.005 0.005 0.005 A3bt
0.048 0.006 0.005 0.002 0.000 0.003 0.003 0.003 0.003 0.003 A3bb/A3bt
0.047 0.006 0.004 0.006 0.003 0.000 0.003 0.002 0.002 0.002 A3/A3g
0.047 0.006 0.004 0.006 0.003 0.003 0.000 0.002 0.002 0.002 A3−A3ad−A3fb
0.046 0.005 0.003 0.005 0.003 0.002 0.002 0.000 0.002 0.002 A3/A3ah
0.046 0.005 0.003 0.005 0.003 0.002 0.002 0.002 0.000 0.002 A3−A3u
0.046 0.005 0.003 0.005 0.003 0.002 0.002 0.002 0.002 0.000 A3−A3fa
A4ac/A3−A4eo
A3ez
A3/A3ez−A3ey
A3bt
A3bb/A3bt
A3/A3g
A3−A3ad−A3fb
A3/A3ah
A3−A3u
A3−A3fa
UniFrac
0.06
0.04
0.02
0
